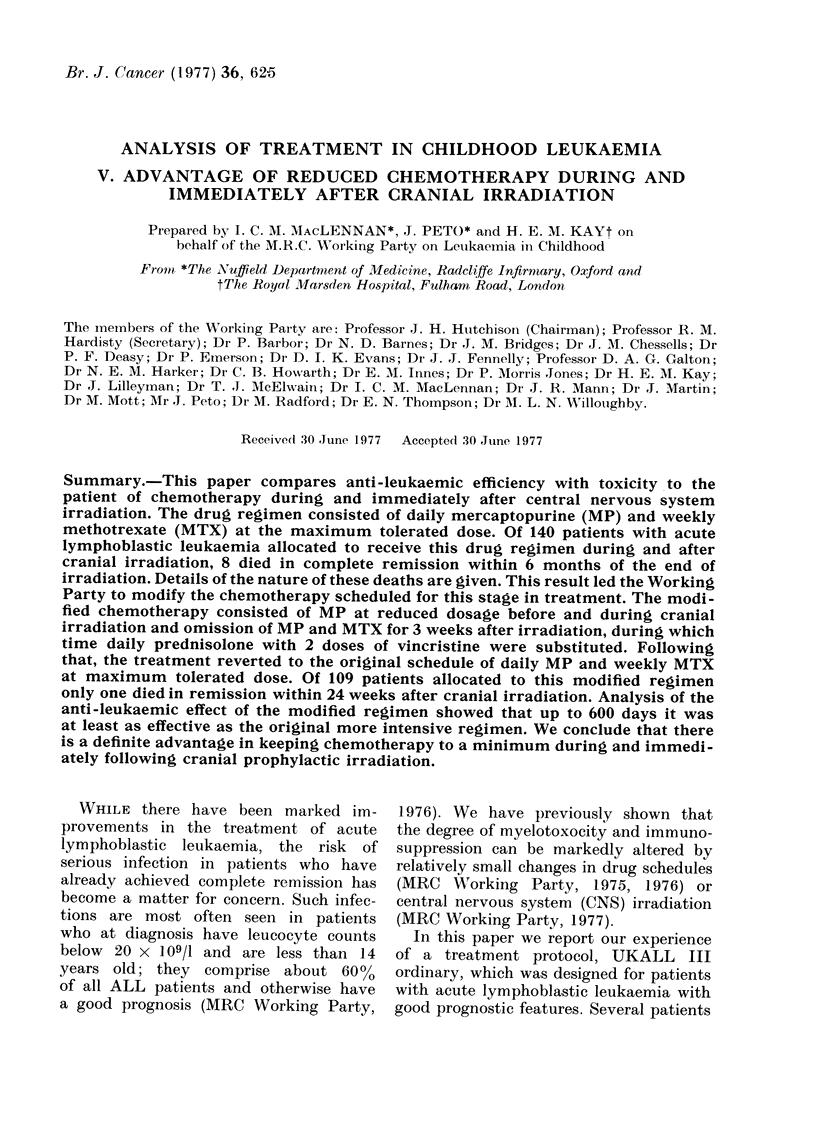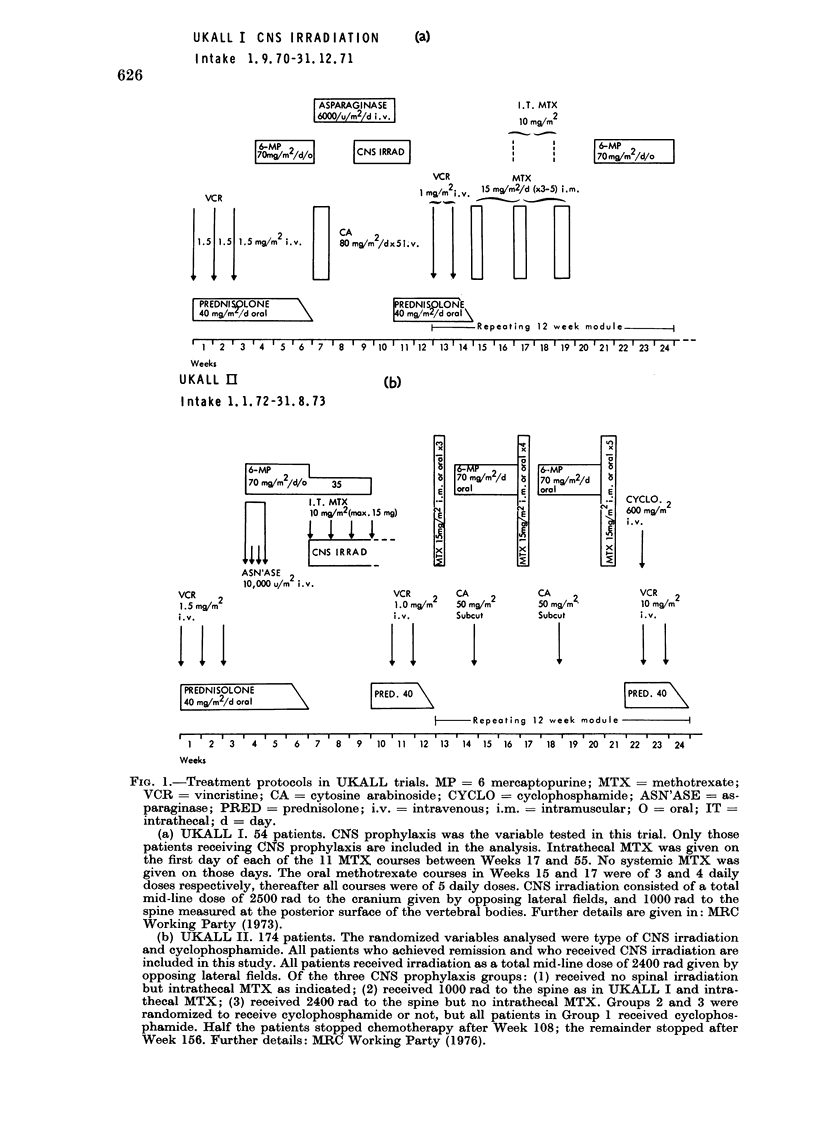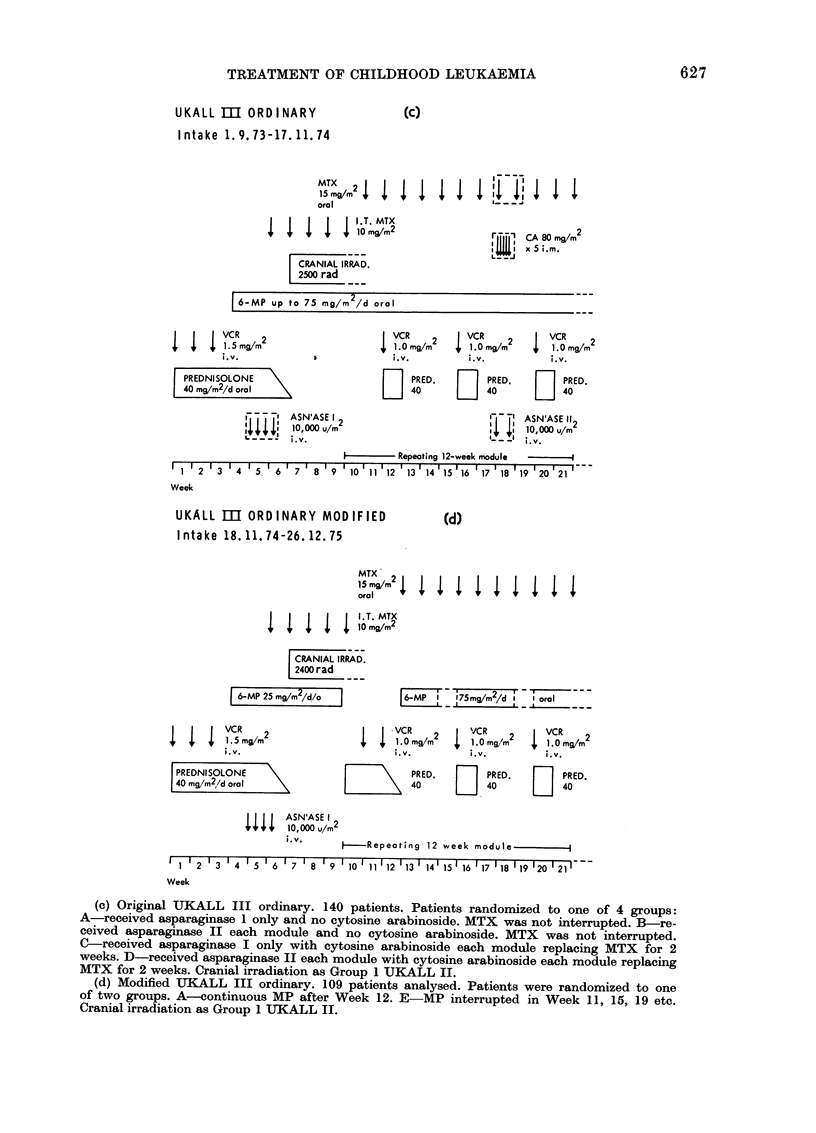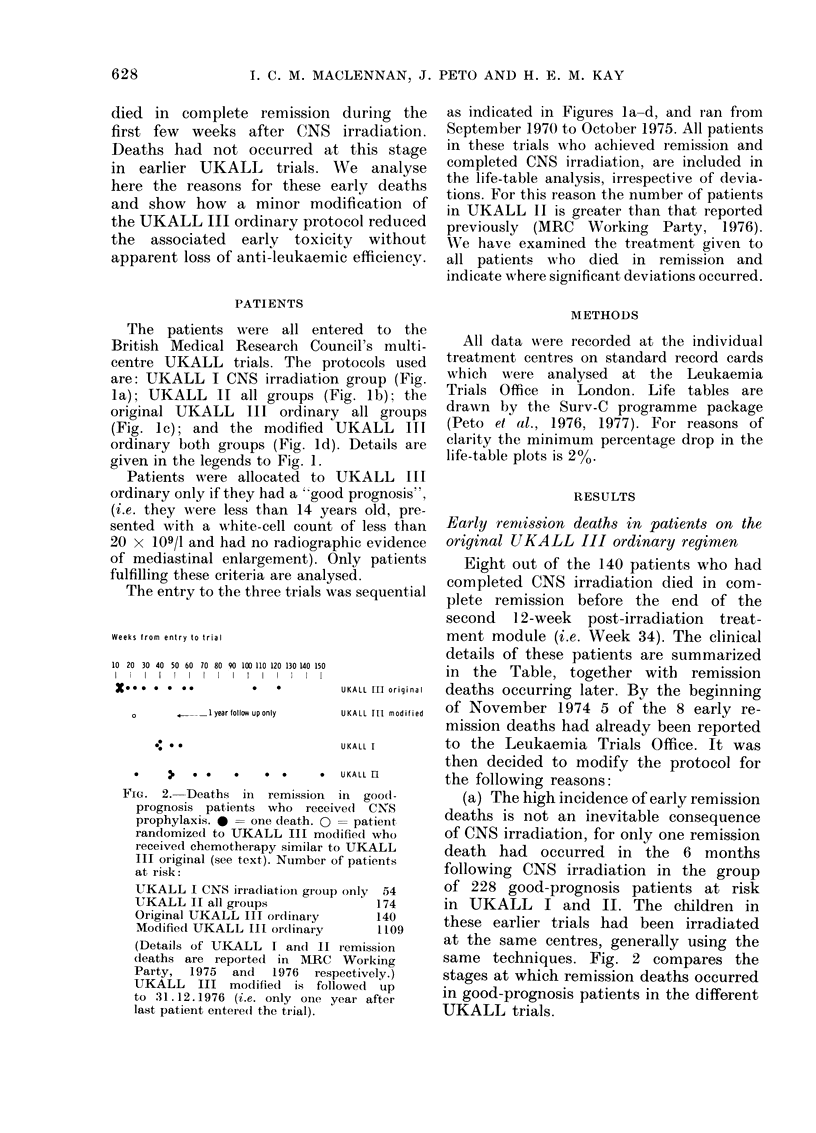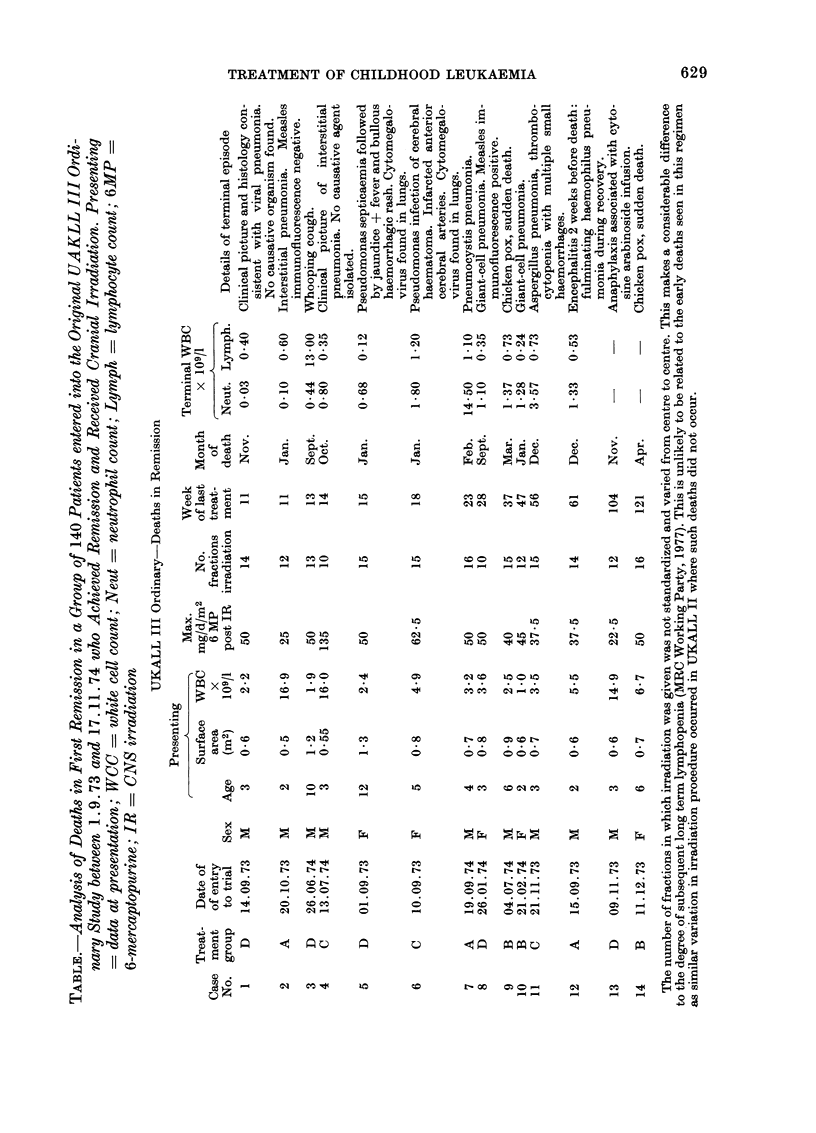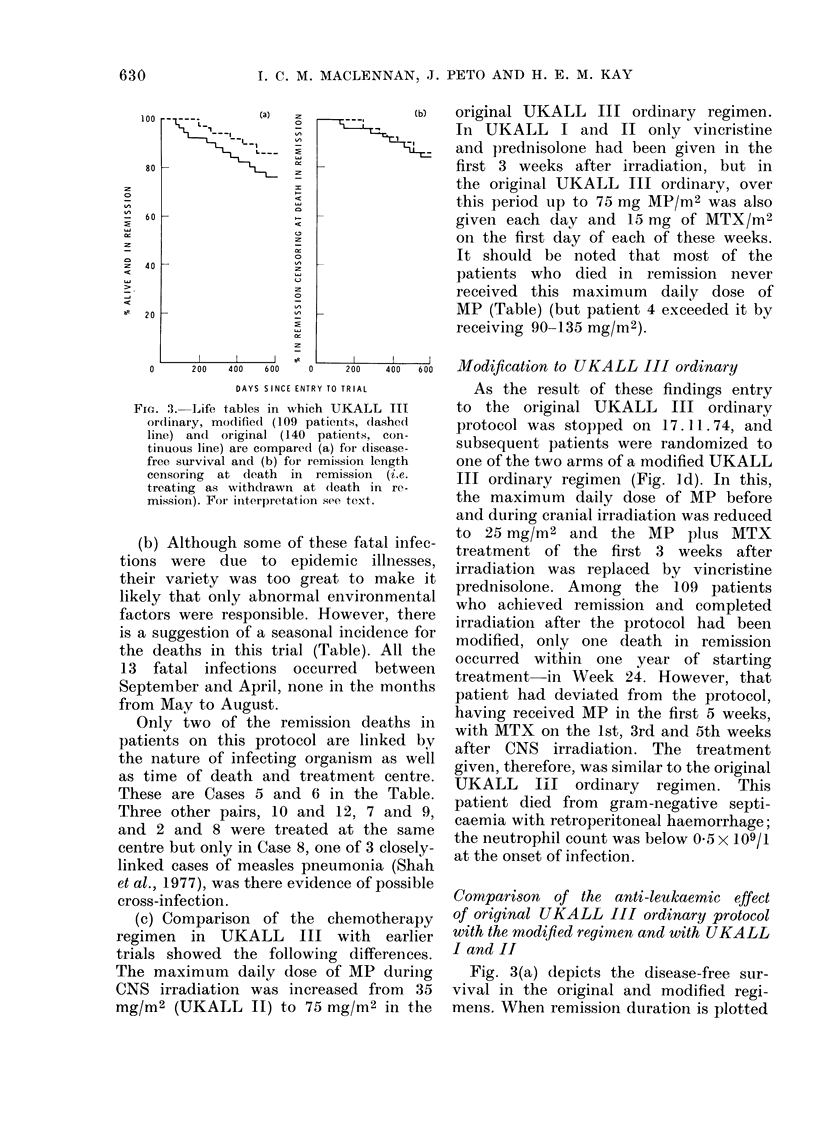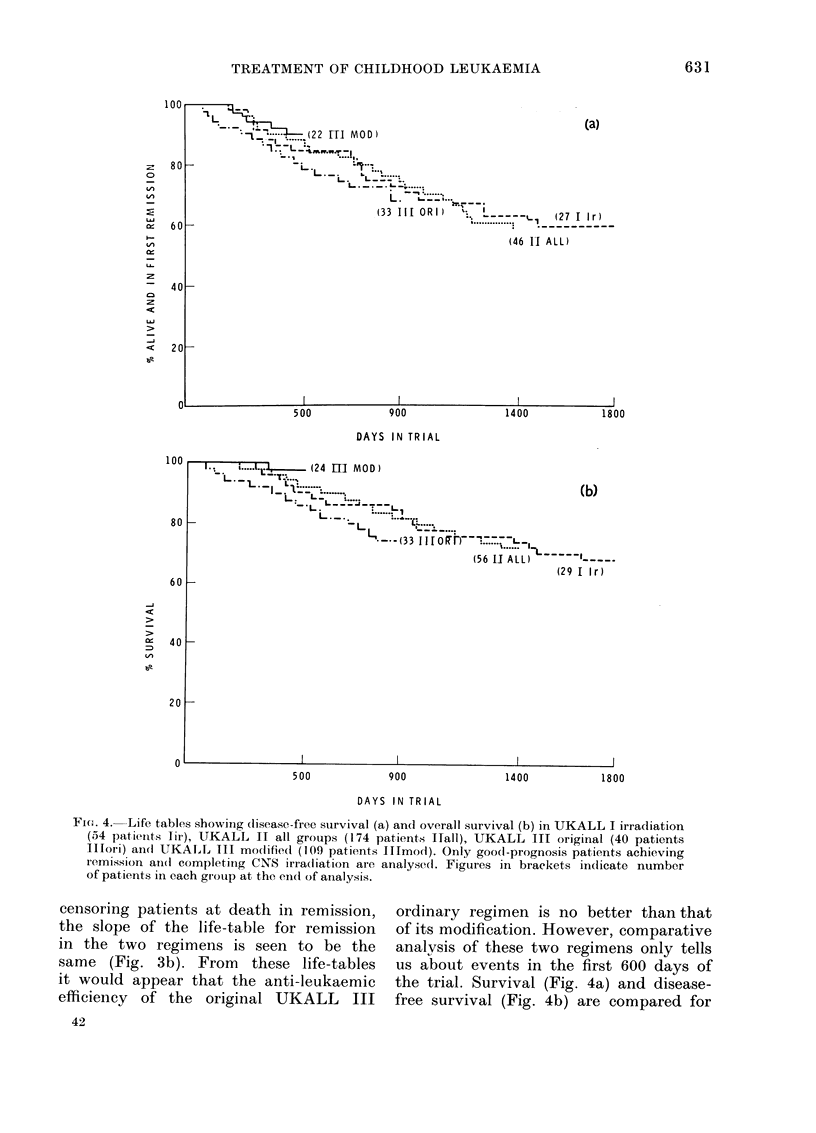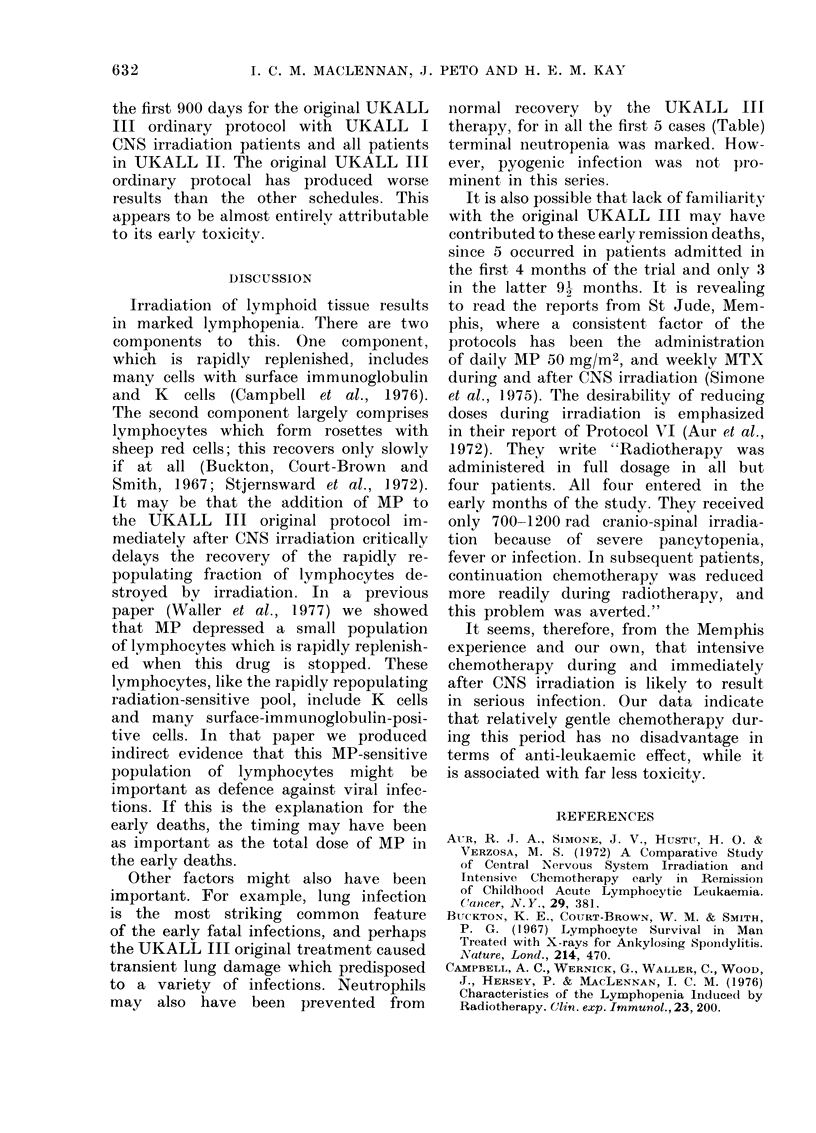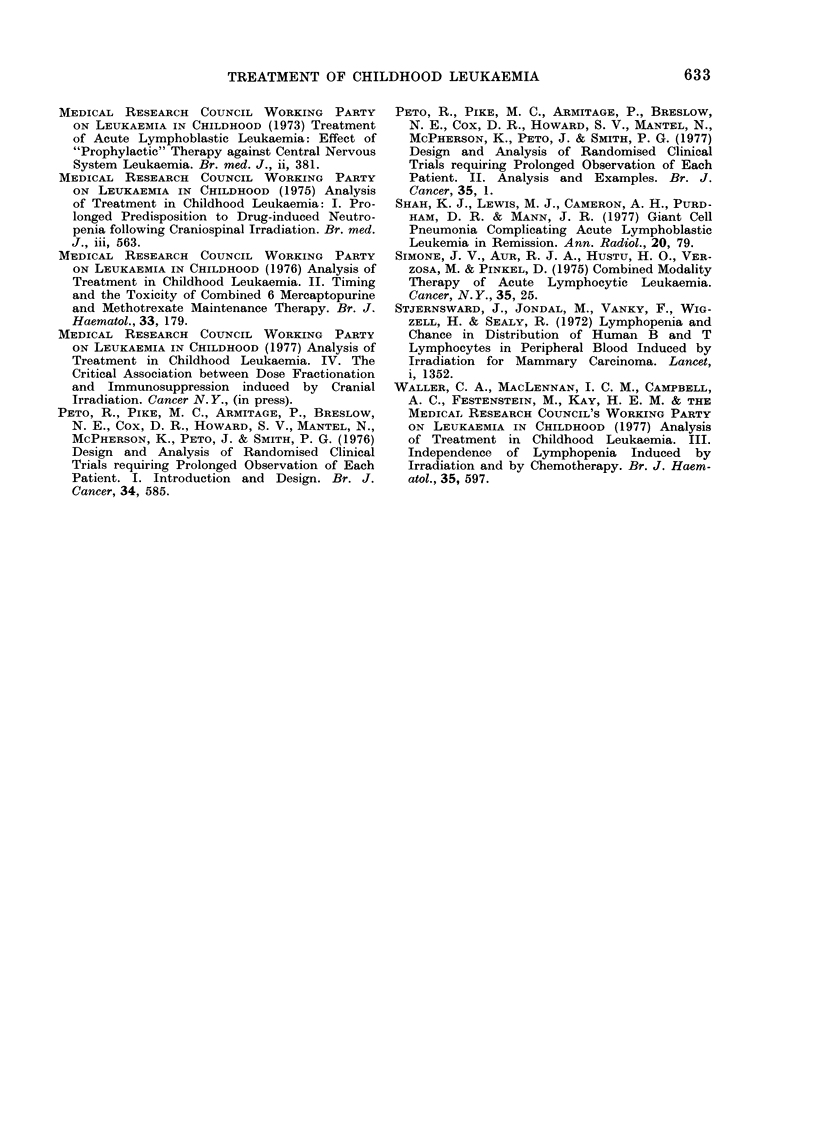# Analysis of treatment of childhood leukaemia. V. Advantage of reduced chemotherapy during and immediately after cranial irradiation.

**DOI:** 10.1038/bjc.1977.240

**Published:** 1977-11

**Authors:** I. C. MacLennan, J. Peto, H. E. Kay

## Abstract

This paper compares anti-leukaemic efficiency with toxicity to the patient of chemotherapy during and immediately after central nervous system irradiation. The drug regimen consisted of daily mercaptopurine (MP) and weekly methotrexate (MTX) at the maximum tolerated dose. Of 140 patients with acute lymphoblastic leukaemia allocated to receive this drug regimen during and after cranial irradiation, 8 died in complete remission within 6 months of the end of irradiation. Details of the nature of these deaths are given. This result led the Working Party to modify the chemotherapy scheduled for this stage in treatment. The modified chemotherapy consisted of MP at reduced dosage before and during cranial irradiation and omission of MP and MTX for 3 weeks after irradiation, during which time daily prednisolone with 2 doses of vincristine were substituted. Following that, the treatment reverted to the original schedule of daily MP and weekly MTX at maximum tolerated dose. Of 109 patients allocated to this modified regimen only one died in remission within 24 weeks after cranial irradiation. Analysis of the anti-leukaemic effect of the modified regimen showed that up to 600 days it was at least as effective as the original more intensive regimen. We conclude that there is a definite advantage in keeping chemotherapy to a minimum during and immediately following cranial prophylactic irradiation.


*Br. J. Cancer* (1977) **36**, 625

### ANALYSIS OF TREATMENT IN CHILDHOOD LEUKAEMIA

### V. ADVANTAGE OF REDUCED CHEMOTHERAPY DURING AND IMMEDIATELY AFTER CRANIAL IRRADIATION

Prepared by I. C. M. MacLENNAN*, J. PETO* and H. E. M. KAY† on behalf of the M.R.C. Working Party on Leukaemia in Childhood

*From *The Nuffield Department of Medicine, Radcliffe Infirmary, Oxford and †The Royal Marsden Hospital, Fulham Road, London*

The members of the Working Party are: Professor J. H. Hutchison (Chairman); Professor R. M. Hardisty (Secretary); Dr P. Barbor; Dr N. D. Barnes; Dr J. M. Bridges; Dr J. M. Chessells; Dr P. F. Deasy; Dr P. Emerson; Dr D. I. K. Evans; Dr J. J. Fennelly; Professor D. A. G. Galton; Dr N. E. M. Harker; Dr C. B. Howarth; Dr E. M. Innes; Dr P. Morris Jones; Dr H. E. M. Kay; Dr J. Lilleyman; Dr T. J. McElwain; Dr I. C. M. MacLennan; Dr J. R. Mann; Dr J. Martin; Dr M. Mott; Mr J. Peto; Dr M. Radford; Dr E. N. Thompson; Dr M. L. N. Willoughby.

Received 30 June 1977 Accepted 30 June 1977

**Summary.—This paper compares anti-leukaemic efficiency with toxicity to the patient of chemotherapy during and immediately after central nervous system irradiation. The drug régimen consisted of daily mercaptopurine (MP) and weekly methotrexate (MTX) at the maximum tolerated dose. Of 140 patients with acute lymphoblastic leukaemia allocated to receive this drug régimen during and after cranial irradiation, 8 died in complete remission within 6 months of the end of irradiation. Details of the nature of these deaths are given. This result led the Working Party to modify the chemotherapy scheduled for this stage in treatment. The modified chemotherapy consisted of MP at reduced dosage before and during cranial irradiation and omission of MP and MTX for 3 weeks after irradiation, during which time daily prednisolone with 2 doses of vincristine were substituted. Following that, the treatment reverted to the original schedule of daily MP and weekly MTX at maximum tolerated dose. Of 109 patients allocated to this modified régimen only one died in remission within 24 weeks after cranial irradiation. Analysis of the anti-leukaemic effect of the modified régimen showed that up to 600 days it was at least as effective as the original more intensive régimen. We conclude that there is a definite advantage in keeping chemotherapy to a minimum during and immediately following cranial prophylactic irradiation.**

While there have been marked improvements in the treatment of acute lymphoblastic leukaemia, the risk of serious infection in patients who have already achieved complete remission has become a matter for concern. Such infections are most often seen in patients who at diagnosis have leucocyte counts below $20 \times 10^9/l$ and are less than 14 years old; they comprise about 60% of all ALL patients and otherwise have a good prognosis (MRC Working Party, 1976). We have previously shown that the degree of myelotoxocity and immunosuppression can be markedly altered by relatively small changes in drug schedules (MRC Working Party, 1975, 1976) or central nervous system (CNS) irradiation (MRC Working Party, 1977).

In this paper we report our experience of a treatment protocol, UKALL III ordinary, which was designed for patients with acute lymphoblastic leukaemia with good prognostic features. Several patients

FIG. 1.—Treatment protocols in UKALL trials. MP = 6 mercaptopurine; MTX = methotrexate; VCR = vincristine; CA = cytosine arabinoside; CYCLO = cyclophosphamide; ASN'ASE = asparaginase; PRED = prednisolone; i.v. = intravenous; i.m. = intramuscular; O = oral; IT = intrathecal; d = day.

(a) UKALL I. 54 patients. CNS prophylaxis was the variable tested in this trial. Only those patients receiving CNS prophylaxis are included in the analysis. Intrathecal MTX was given on the first day of each of the 11 MTX courses between Weeks 17 and 55. No systemic MTX was given on those days. The oral methotrexate courses in Weeks 15 and 17 were of 3 and 4 daily doses respectively, thereafter all courses were of 5 daily doses. CNS irradiation consisted of a total mid-line dose of 2500 rad to the cranium given by opposing lateral fields, and 1000 rad to the spine measured at the posterior surface of the vertebral bodies. Further details are given in: MRC Working Party (1973).

(b) UKALL II. 174 patients. The randomized variables analysed were type of CNS irradiation and cyclophosphamide. All patients who achieved remission and who received CNS irradiation are included in this study. All patients received irradiation as a total mid-line dose of 2400 rad given by opposing lateral fields. Of the three CNS prophylaxis groups: (1) received no spinal irradiation but intrathecal MTX as indicated; (2) received 1000 rad to the spine as in UKALL I and intrathecal MTX; (3) received 2400 rad to the spine but no intrathecal MTX. Groups 2 and 3 were randomized to receive cyclophosphamide or not, but all patients in Group 1 received cyclophosphamide. Half the patients stopped chemotherapy after Week 108; the remainder stopped after Week 156. Further details: MRC Working Party (1976).

UKALL III ORDINARY (c)

Intake 1.9.73-17.11.74

UKALL III ORDINARY MODIFIED (d)

Intake 18.11.74-26.12.75

(c) Original UKALL III ordinary. 140 patients. Patients randomized to one of 4 groups: A—received asparaginase 1 only and no cytosine arabinoside. MTX was not interrupted. B—received asparaginase II each module and no cytosine arabinoside. MTX was not interrupted. C—received asparaginase I only with cytosine arabinoside each module replacing MTX for 2 weeks. D—received asparaginase II each module with cytosine arabinoside each module replacing MTX for 2 weeks. Cranial irradiation as Group 1 UKALL II.

(d) Modified UKALL III ordinary. 109 patients analysed. Patients were randomized to one of two groups. A—continuous MP after Week 12. E—MP interrupted in Week 11, 15, 19 etc. Cranial irradiation as Group 1 UKALL II.

died in complete remission during the first few weeks after CNS irradiation. Deaths had not occurred at this stage in earlier UKALL trials. We analyse here the reasons for these early deaths and show how a minor modification of the UKALL III ordinary protocol reduced the associated early toxicity without apparent loss of anti-leukaemic efficiency.

### PATIENTS

The patients were all entered to the British Medical Research Council's multi-centre UKALL trials. The protocols used are: UKALL I CNS irradiation group (Fig. 1a); UKALL II all groups (Fig. 1b); the original UKALL III ordinary all groups (Fig. 1c); and the modified UKALL III ordinary both groups (Fig. 1d). Details are given in the legends to Fig. 1.

Patients were allocated to UKALL III ordinary only if they had a "good prognosis", (*i.e.* they were less than 14 years old, presented with a white-cell count of less than $20 \times 10^9$/l and had no radiographic evidence of mediastinal enlargement). Only patients fulfilling these criteria are analysed.

The entry to the three trials was sequential as indicated in Figures 1a–d, and ran from September 1970 to October 1975. All patients in these trials who achieved remission and completed CNS irradiation, are included in the life-table analysis, irrespective of deviations. For this reason the number of patients in UKALL II is greater than that reported previously (MRC Working Party, 1976). We have examined the treatment given to all patients who died in remission and indicate where significant deviations occurred.

FIG. 2.—Deaths in remission in good-prognosis patients who received CNS prophylaxis. ● = one death. ○ = patient randomized to UKALL III modified who received chemotherapy similar to UKALL III original (see text). Number of patients at risk:

| | |
|---|---|
| UKALL I CNS irradiation group only | 54 |
| UKALL II all groups | 174 |
| Original UKALL III ordinary | 140 |
| Modified UKALL III ordinary | 1109 |

(Details of UKALL I and II remission deaths are reported in MRC Working Party, 1975 and 1976 respectively.) UKALL III modified is followed up to 31.12.1976 (*i.e.* only one year after last patient entered the trial).

### METHODS

All data were recorded at the individual treatment centres on standard record cards which were analysed at the Leukaemia Trials Office in London. Life tables are drawn by the Surv-C programme package (Peto *et al.*, 1976, 1977). For reasons of clarity the minimum percentage drop in the life-table plots is 2%.

### RESULTS

*Early remission deaths in patients on the original UKALL III ordinary regimen*

Eight out of the 140 patients who had completed CNS irradiation died in complete remission before the end of the second 12-week post-irradiation treatment module (*i.e.* Week 34). The clinical details of these patients are summarized in the Table, together with remission deaths occurring later. By the beginning of November 1974 5 of the 8 early remission deaths had already been reported to the Leukaemia Trials Office. It was then decided to modify the protocol for the following reasons:

(a) The high incidence of early remission deaths is not an inevitable consequence of CNS irradiation, for only one remission death had occurred in the 6 months following CNS irradiation in the group of 228 good-prognosis patients at risk in UKALL I and II. The children in these earlier trials had been irradiated at the same centres, generally using the same techniques. Fig. 2 compares the stages at which remission deaths occurred in good-prognosis patients in the different UKALL trials.

FIG. 3.—Life tables in which UKALL III ordinary, modified (109 patients, dashed line) and original (140 patients, continuous line) are compared (a) for disease-free survival and (b) for remission length censoring at death in remission (*i.e.* treating as withdrawn at death in remission). For interpretation see text.

(b) Although some of these fatal infections were due to epidemic illnesses, their variety was too great to make it likely that only abnormal environmental factors were responsible. However, there is a suggestion of a seasonal incidence for the deaths in this trial (Table). All the 13 fatal infections occurred between September and April, none in the months from May to August.

Only two of the remission deaths in patients on this protocol are linked by the nature of infecting organism as well as time of death and treatment centre. These are Cases 5 and 6 in the Table. Three other pairs, 10 and 12, 7 and 9, and 2 and 8 were treated at the same centre but only in Case 8, one of 3 closely-linked cases of measles pneumonia (Shah *et al.*, 1977), was there evidence of possible cross-infection.

(c) Comparison of the chemotherapy regimen in UKALL III with earlier trials showed the following differences. The maximum daily dose of MP during CNS irradiation was increased from 35 mg/m$^2$ (UKALL II) to 75 mg/m$^2$ in the original UKALL III ordinary regimen. In UKALL I and II only vincristine and prednisolone had been given in the first 3 weeks after irradiation, but in the original UKALL III ordinary, over this period up to 75 mg MP/m$^2$ was also given each day and 15 mg of MTX/m$^2$ on the first day of each of these weeks. It should be noted that most of the patients who died in remission never received this maximum daily dose of MP (Table) (but patient 4 exceeded it by receiving 90–135 mg/m$^2$).

*Modification to UKALL III ordinary*

As the result of these findings entry to the original UKALL III ordinary protocol was stopped on 17.11.74, and subsequent patients were randomized to one of the two arms of a modified UKALL III ordinary regimen (Fig. 1d). In this, the maximum daily dose of MP before and during cranial irradiation was reduced to 25 mg/m$^2$ and the MP plus MTX treatment of the first 3 weeks after irradiation was replaced by vincristine prednisolone. Among the 109 patients who achieved remission and completed irradiation after the protocol had been modified, only one death in remission occurred within one year of starting treatment—in Week 24. However, that patient had deviated from the protocol, having received MP in the first 5 weeks, with MTX on the 1st, 3rd and 5th weeks after CNS irradiation. The treatment given, therefore, was similar to the original UKALL III ordinary regimen. This patient died from gram-negative septicaemia with retroperitoneal haemorrhage; the neutrophil count was below $0{\cdot}5 \times 10^9/l$ at the onset of infection.

*Comparison of the anti-leukaemic effect of original UKALL III ordinary protocol with the modified regimen and with UKALL I and II*

Fig. 3(a) depicts the disease-free survival in the original and modified regimens. When remission duration is plotted

FIG. 4.—Life tables showing disease-free survival (a) and overall survival (b) in UKALL I irradiation (54 patients Iir), UKALL II all groups (174 patients IIall), UKALL III original (40 patients IIIori) and UKALL III modified (109 patients IIImod). Only good-prognosis patients achieving remission and completing CNS irradiation are analysed. Figures in brackets indicate number of patients in each group at the end of analysis.

censoring patients at death in remission, the slope of the life-table for remission in the two regimens is seen to be the same (Fig. 3b). From these life-tables it would appear that the anti-leukaemic efficiency of the original UKALL III ordinary regimen is no better than that of its modification. However, comparative analysis of these two regimens only tells us about events in the first 600 days of the trial. Survival (Fig. 4a) and disease-free survival (Fig. 4b) are compared for the first 900 days for the original UKALL III ordinary protocol with UKALL I CNS irradiation patients and all patients in UKALL II. The original UKALL III ordinary protocal has produced worse results than the other schedules. This appears to be almost entirely attributable to its early toxicity.

### DISCUSSION

Irradiation of lymphoid tissue results in marked lymphopenia. There are two components to this. One component, which is rapidly replenished, includes many cells with surface immunoglobulin and K cells (Campbell *et al.*, 1976). The second component largely comprises lymphocytes which form rosettes with sheep red cells; this recovers only slowly if at all (Buckton, Court-Brown and Smith, 1967; Stjernsward *et al.*, 1972). It may be that the addition of MP to the UKALL III original protocol immediately after CNS irradiation critically delays the recovery of the rapidly repopulating fraction of lymphocytes destroyed by irradiation. In a previous paper (Waller *et al.*, 1977) we showed that MP depressed a small population of lymphocytes which is rapidly replenished when this drug is stopped. These lymphocytes, like the rapidly repopulating radiation-sensitive pool, include K cells and many surface-immunoglobulin-positive cells. In that paper we produced indirect evidence that this MP-sensitive population of lymphocytes might be important as defence against viral infections. If this is the explanation for the early deaths, the timing may have been as important as the total dose of MP in the early deaths.

Other factors might also have been important. For example, lung infection is the most striking common feature of the early fatal infections, and perhaps the UKALL III original treatment caused transient lung damage which predisposed to a variety of infections. Neutrophils may also have been prevented from normal recovery by the UKALL III therapy, for in all the first 5 cases (Table) terminal neutropenia was marked. However, pyogenic infection was not prominent in this series.

It is also possible that lack of familiarity with the original UKALL III may have contributed to these early remission deaths, since 5 occurred in patients admitted in the first 4 months of the trial and only 3 in the latter $9\frac{1}{2}$ months. It is revealing to read the reports from St Jude, Memphis, where a consistent factor of the protocols has been the administration of daily MP 50 $mg/m^2$, and weekly MTX during and after CNS irradiation (Simone *et al.*, 1975). The desirability of reducing doses during irradiation is emphasized in their report of Protocol VI (Aur *et al.*, 1972). They write "Radiotherapy was administered in full dosage in all but four patients. All four entered in the early months of the study. They received only 700–1200 rad cranio-spinal irradiation because of severe pancytopenia, fever or infection. In subsequent patients, continuation chemotherapy was reduced more readily during radiotherapy, and this problem was averted."

It seems, therefore, from the Memphis experience and our own, that intensive chemotherapy during and immediately after CNS irradiation is likely to result in serious infection. Our data indicate that relatively gentle chemotherapy during this period has no disadvantage in terms of anti-leukaemic effect, while it is associated with far less toxicity.

### REFERENCES

Aur, R. J. A., Simone, J. V., Hustu, H. O. & Verzosa, M. S. (1972) A Comparative Study of Central Nervous System Irradiation and Intensive Chemotherapy early in Remission of Childhood Acute Lymphocytic Leukaemia. *Cancer, N.Y.*, **29**, 381.

Buckton, K. E., Court-Brown, W. M. & Smith, P. G. (1967) Lymphocyte Survival in Man Treated with X-rays for Ankylosing Spondylitis. *Nature, Lond.*, **214**, 470.

Campbell, A. C., Wernick, G., Waller, C., Wood, J., Hersey, P. & MacLennan, I. C. M. (1976) Characteristics of the Lymphopenia Induced by Radiotherapy. *Clin. exp. Immunol.*, **23**, 200.

MEDICAL RESEARCH COUNCIL WORKING PARTY ON LEUKAEMIA IN CHILDHOOD (1973) Treatment of Acute Lymphoblastic Leukaemia: Effect of "Prophylactic" Therapy against Central Nervous System Leukaemia. *Br. med. J.*, ii, 381.

MEDICAL RESEARCH COUNCIL WORKING PARTY ON LEUKAEMIA IN CHILDHOOD (1975) Analysis of Treatment in Childhood Leukaemia: I. Prolonged Predisposition to Drug-induced Neutropenia following Craniospinal Irradiation. *Br. med. J.*, iii, 563.

MEDICAL RESEARCH COUNCIL WORKING PARTY ON LEUKAEMIA IN CHILDHOOD (1976) Analysis of Treatment in Childhood Leukaemia. II. Timing and the Toxicity of Combined 6 Mercaptopurine and Methotrexate Maintenance Therapy. *Br. J. Haematol.*, **33**, 179.

MEDICAL RESEARCH COUNCIL WORKING PARTY ON LEUKAEMIA IN CHILDHOOD (1977) Analysis of Treatment in Childhood Leukaemia. IV. The Critical Association between Dose Fractionation and Immunosuppression induced by Cranial Irradiation. *Cancer N.Y.*, (in press).

PETO, R., PIKE, M. C., ARMITAGE, P., BRESLOW, N. E., COX, D. R., HOWARD, S. V., MANTEL, N., MCPHERSON, K., PETO, J. & SMITH, P. G. (1976) Design and Analysis of Randomised Clinical Trials requiring Prolonged Observation of Each Patient. I. Introduction and Design. *Br. J. Cancer*, **34**, 585.

PETO, R., PIKE, M. C., ARMITAGE, P., BRESLOW, N. E., COX, D. R., HOWARD, S. V., MANTEL, N., MCPHERSON, K., PETO, J. & SMITH, P. G. (1977) Design and Analysis of Randomised Clinical Trials requiring Prolonged Observation of Each Patient. II. Analysis and Examples. *Br. J. Cancer*, **35**, 1.

SHAH, K. J., LEWIS, M. J., CAMERON, A. H., PURDHAM, D. R. & MANN, J. R. (1977) Giant Cell Pneumonia Complicating Acute Lymphoblastic Leukemia in Remission. *Ann. Radiol.*, **20**, 79.

SIMONE, J. V., AUR, R. J. A., HUSTU, H. O., VERZOSA, M. & PINKEL, D. (1975) Combined Modality Therapy of Acute Lymphocytic Leukaemia. *Cancer, N.Y.*, **35**, 25.

STJERNSWARD, J., JONDAL, M., VANKY, F., WIGZELL, H. & SEALY, R. (1972) Lymphopenia and Chance in Distribution of Human B and T Lymphocytes in Peripheral Blood Induced by Irradiation for Mammary Carcinoma. *Lancet*, i, 1352.

WALLER, C. A., MACLENNAN, I. C. M., CAMPBELL, A. C., FESTENSTEIN, M., KAY, H. E. M. & THE MEDICAL RESEARCH COUNCIL'S WORKING PARTY ON LEUKAEMIA IN CHILDHOOD (1977) Analysis of Treatment in Childhood Leukaemia. III. Independence of Lymphopenia Induced by Irradiation and by Chemotherapy. *Br. J. Haematol.*, **35**, 597.